# Mitochondrial damage and post-translational modifications in radiation-induced heart disease: mechanisms and emerging therapeutic targets

**DOI:** 10.3389/fped.2026.1914384

**Published:** 2026-08-20

**Authors:** Na Zuo, Yaling Li, Jun Yang

**Affiliations:** 1 Department of Cardiology, The First Affiliated Hospital, Hengyang Medical School, University of South China, Hengyang, Hunan, China; 2 Hunan Provincial Key Laboratory of Multi-omics and Artificial Intelligence of Cardiovascular Diseases, University of South China, Hengyang, Hunan, China; 3 Clinical Research Center for Myocardial Injury in Hunan Province, Hengyang, Hunan, China

**Keywords:** mitochondrial damage, mitophagy, post-translational modifications, radiation-induced heart disease, therapeutic targets

## Abstract

**Background:**

Radiation-induced heart disease (RIHD) is a major late complication of thoracic radiotherapy. However, the mechanisms responsible for its long-term progression remain poorly understood. Conventional explanations, such as DNA damage, and oxidative stress, mainly focus on early radiation responses and fail to fully account for the prolonged latency and progressive myocardial remodeling observed in RIHD over years to decades.

**Main content:**

This review proposes that persistent disruption of mitochondrial homeostasis represents a central link between early radiation-induced injury and late cardiac remodeling. We systematically summarize the major processes involved in radiation-induced mitochondrial dysfunction. These processes include mtDNA damage, respiratory chain impairment, sustained mitochondrial reactive oxygen species (mitoROS) accumulation, metabolic network remodeling, and defective mitochondrial clearance through mitophagy. We further discuss how acetylation, SUMOylation, and lactylation regulate these processes and contribute to the development and progression of RIHD. Building on this framework, we highlight emerging molecular targets, including NDP52, ATP5F1C, P4HB, and SH3GLB1, as well as the potential protective effects of bioactive compounds derived from traditional Chinese medicine, such as aloe-emodin and astragaloside IV, through restoration of mitochondrial homeostasis. By integrating mitochondrial dysfunction, post-translational modifications, and mitophagy into a unified pathological framework, this review provides new perspectives for early identification and therapeutic intervention in RIHD.

**Key conclusions:**

RIHD is not simply an oxidative stress-driven disorder but rather a chronic remodeling process shaped by the interplay among mitochondrial injury, post-translational modifications, and mitophagy dysregulation. This integrated framework links early subcellular alterations to late cardiac remodeling and provides a potential biological explanation for the long latency and progressive nature of RIHD. However, most current evidence is derived from adult models. Future research should specifically address childhood cancer survivors, as radiation exposure during critical periods of cardiac development may result in distinct long-term cardiovascular outcomes.

## 1 Introduction

Radiotherapy plays an essential role in treating thoracic malignancies, including breast cancer, lung cancer, esophageal cancer, Hodgkin lymphoma, and mediastinal tumors. As radiotherapy techniques improve and cancer survival increases, cardiovascular complications have become major factors affecting survivors' long-term quality of life and non-cancer mortality. Clinical evidence shows that cardiac radiation exposure is closely associated with subsequent cardiovascular events. A landmark study by Darby et al. found a linear association between the mean heart dose and the risk of major coronary events in patients with breast cancer. The risk increased by 7.4% for each 1 Gy increase in the mean heart dose (1). A long-term follow-up study by van Nimwegen et al. showed that Hodgkin lymphoma survivors remained at increased risks of coronary artery disease, valvular heart disease, and heart failure for more than 30 years after treatment. These findings highlight the persistent and progressive nature of Radiation-induced heart disease (RIHD) (2).

Importantly, RIHD is not limited to patients who receive high-dose thoracic radiotherapy. Epidemiological studies of atomic bomb survivors in Hiroshima and participants in the International Nuclear Workers Study have linked low-dose or chronic occupational radiation exposure to an increased cardiovascular risk (3, 4). These findings suggest that radiation-induced cardiovascular injury may not have an absolute dose threshold. RIHD is now recognized as a complex syndrome with diverse cardiovascular manifestations. These manifestations include coronary artery disease, microvascular dysfunction, myocardial fibrosis, pericardial disease, valvular abnormalities, conduction disturbances, and heart failure (5). Traditionally, researchers have attributed radiation-induced cardiac injury to DNA damage, oxidative stress, endothelial dysfunction, and inflammatory responses (6, 7). However, these mechanisms do not fully explain the long latency and progressive cardiac remodeling associated with RIHD. The disease often develops over many years rather than presenting as acute radiation toxicity. This knowledge gap has drawn attention to more complex molecular networks. These networks involve mitochondrial dysfunction, metabolic reprogramming, epigenetic regulation, and post-translational modifications (PTMs), which may jointly drive the onset and progression of RIHD (8).

Cardiomyocytes rely heavily on mitochondrial oxidative phosphorylation for energy. Therefore, mitochondrial homeostasis is essential for normal cardiac function. Radiation can damage mitochondrial DNA and impair the electron transport chain. These changes cause ATP depletion and cause persistent accumulation of mitochondrial reactive oxygen species, which further disrupt mitochondrial dynamics and mitophagy (7, 8). Growing evidence suggests that mitochondrial dysfunction alone may not be the central issue in RIHD. The ability of cells to recognize, engulf, and deliver damaged mitochondria to lysosomes for degradation may be equally important. Proper mitophagy removes dysfunctional mitochondria and limits the spread of mitochondrial reactive oxygen species. In contrast, persistent mitophagy-related signaling or incomplete mitochondrial degradation may worsen oxidative stress and promote fibrotic remodeling.

PTMs have emerged as key regulators of mitochondrial quality control. They regulate mitochondrial metabolism and mitophagy at multiple levels. Acetylation modulates mitochondrial bioenergetics. SUMOylation controls the recognition of damaged mitochondria and the recruitment of autophagic machinery (9). Lactylation connects metabolic reprogramming with mitochondrial quality control. Therefore, this review first outlines the general role of mitochondrial dysfunction in RIHD. It then focuses on the PTM-mediated network that regulate mitophagy and mitochondrial metabolism. In particular, we examine ATP5F1C acetylation, NDP52/SH3GLB1 SUMOylation, and P4HB lactylation. These molecular events may serve as key mechanistic links between mitochondrial injury and chronic cardiac remodeling.

Importantly, this framework is especially relevant to childhood cancer survivors. In these patients, radiation exposure occurs during a critical period of cardiac development and metabolic maturation. However, most current mechanistic evidence comes from adult animal models. It remains unclear whether the developing heart responds differently to radiation-induced mitochondrial injury (10).

**Figure 1:**
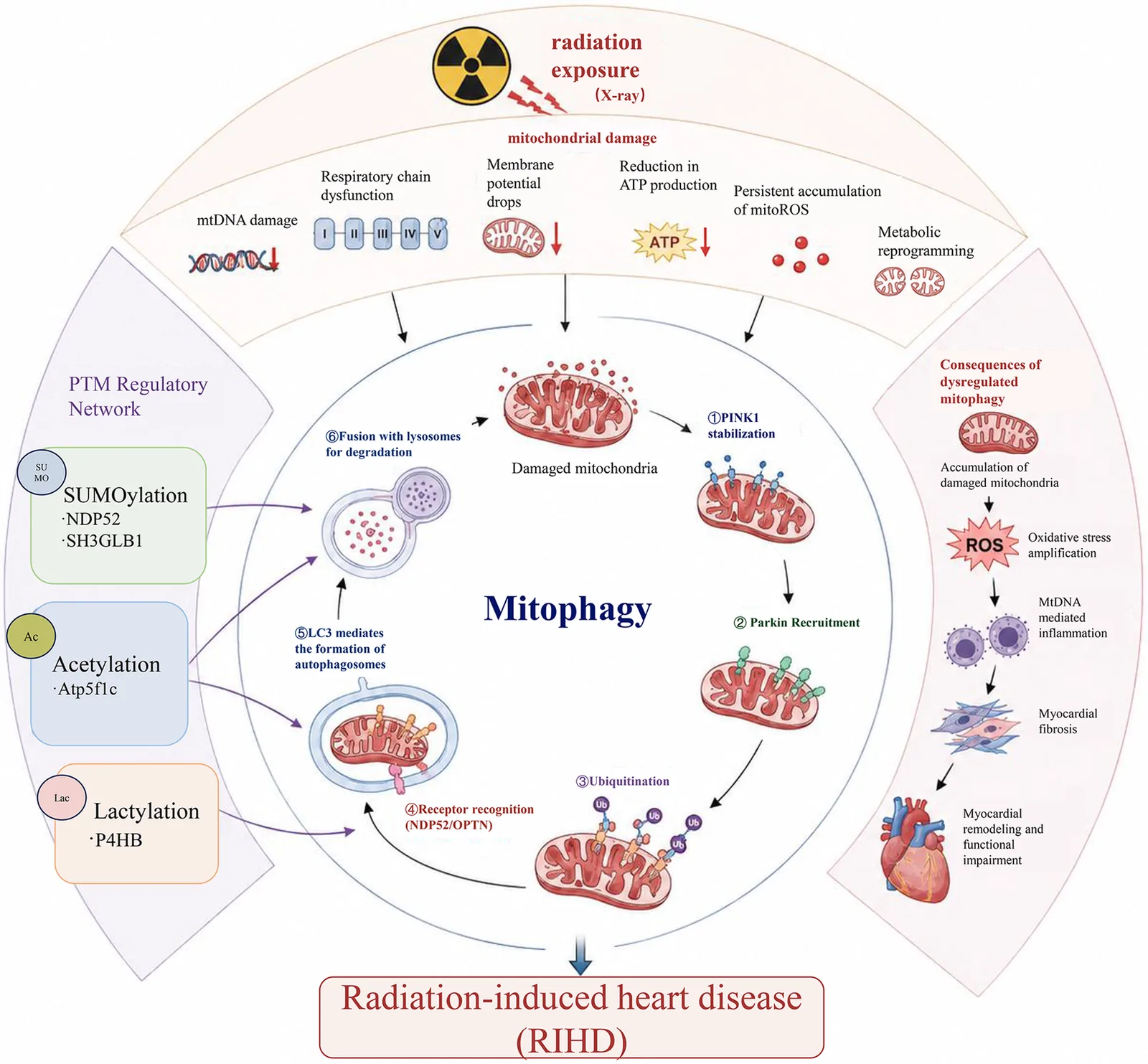
Schematic overview of mitochondrial injury, PTMs, and mitophagy dysregulation in RIHD. Ionizing radiation (X-ray) triggers mitochondrial damage, characterized by mtDNA lesions, respiratory chain dysfunction, ATP depletion, and persistent accumulation of mitochondrial reactive oxygen species (mitoROS). These insults induce metabolic reprogramming and initiate mitophagy. The efficiency and outcome of mitophagy are critically modulated by post-translational modifications (PTMs), specifically SUMOylation of NDP52 and SH3GLB1, acetylation of ATP5F1C, and lactylation of P4HB. Dysregulated mitophagy leads to the accumulation of damaged mitochondria, exacerbating oxidative stress and activating inflammatory responses, which collectively promote myocardial fibrosis and adverse cardiac remodeling in radiation-induced heart disease (RIHD).

To clarify the mechanisms discussed in this review, Figure 1 presents a schematic overview of RIHD. The diagram summarizes the cascade of radiation-induced mitochondrial injury, PTM-mediated regulation, and mitophagy dysregulation.

## 2 The central role of mitochondria in cardiomyocytes

The heart is one of the most energy-demanding organs in the body. Its continuous contractile activity requires a stable and efficient energy supply. In adult cardiomyocytes, mitochondria occupy approximately 30%–40% of the cell volume. They generate more than 90% of cellular ATP through oxidative phosphorylation. This ATP production provides the energy needed to maintain normal cardiac pump function (11, 12). Under physiological conditions, the heart primarily utilizes fatty acid β-oxidation for energy. It can also switch to glucose or lactate oxidation according to substrate availability, and physiological demands. This metabolic flexibility allows the heart to adapt to changes in nutrient supply and various forms of stress (12).

Beyond ATP production, mitochondria regulate excitation–contraction coupling in cardiomyocytes through calcium uptake and release. Mitochondria take up and release calcium in accordance with each heartbeat. Moderate mitochondrial calcium uptake activates several dehydrogenases in the tricarboxylic acid cycle and promotes ATP synthesis. This mechanism allows energy contraction to match the demands of cardiac contraction (13). However, excessive mitochondrial calcium accumulation can promote mitochondrial dysfunction and cellular injury. In addition, the electron transport chain continuously produces small amounts of reactive oxygen species (ROS). At physiological levels, ROS act as second messengers that regulate multiple signaling pathways and help maintain normal cellular function (14).

Normal mitochondrial function depends on a highly coordinated mitochondrial quality control system. This system maintains a dynamic balance among mitochondrial fusion and fission, mitochondrial biogenesis, and mitophagy. Mitochondrial fusion allows mitochondria to exchange their contents and complement each other's functions. This process helps maintain the integrity of the mitochondrial network. In contrast, mitochondrial fission segregates damaged or dysfunctional segments from the network and prepares them for selective removal through mitophagy. Cardiomyocytes are terminally differentiated cells with very limited proliferative capacity (15–17). Therefore, they cannot dilute or replace damaged mitochondria through cell division. Maintaining mitochondrial homeostasis is thus essential for normal cardiac function and may strongly influence the onset and progression of heart disease.

## 3 Radiation-induced mitochondrial dysfunction in RIHD

### 3.1 Mitochondrial DNA damage and persistent mitochondrial reactive oxygen species amplification

Mitochondrial DNA (mtDNA) damage is an early and critical event in radiation-induced mitochondrial dysfunction. Its effects may persist long after radiation exposure. mtDNA encodes key components of the oxidative phosphorylation system and is essential for the assembly and function of respiratory-chain complexes. mtDNA is located within the highly metabolically active environment of mitochondria. Compared with nuclear DNA, mtDNA has less protective packaging and more limited repair capacity. These features make mtDNA particularly vulnerable to radiation and subsequent oxidative stress (18, 19).

Ionizing radiation produces free radicals through direct ionization and water radiolysis. It can also damage mtDNA, mitochondrial membranes, and respiratory-chain proteins (20). Barjaktarovic et al. examined cardiac radiation injury in a C57BL/6 mouse model. At four weeks after exposure to 2 Gy, the irradiated hearts showed altered expression of proteins involved in oxidative phosphorylation, and pyruvate metabolism. The researchers also observed impaired succinate-driven respiration, and increased mitochondrial protein oxidation (21). The same group later reported that some respiratory abnormalities persisted for up to 40 weeks after irradiation (22). Sridharan et al. also observed impaired in mitochondrial membrane integrity and respiratory function in irradiated rat hearts (23). These findings indicate that radiation not only triggers an acute free-radical response. It also causes long-lasting structural and functional abnormalities in cardiac mitochondria.

In addition, mtDNA damage may convert mitochondrial metabolic disturbances into inflammatory signals. Following mitochondrial injury, mtDNA released into the cytoplasm acts as a damage-associated molecular pattern (DAMP) and triggers innate immune responses. Zhong et al. identified mtDNA as an important driver of chronic inflammatory diseases (24). Furthermore, Philipp et al. demonstrated that the cyclic GMP–AMP synthase-stimulator of interferon genes (cGAS-STING) pathway plays a central role in radiation responses in human cardiac endothelial cells (25). These findings suggest that mitochondrial dysfunction not only impairs cellular energy metabolism but also contributes to sterile inflammation in RIHD through mtDNA release and innate immune activation.

Mitochondrial dysfunction, telomere abnormalities, and cellular senescence may reinforce one another. Using a chemoptogenetic approach, Qian et al. showed that selective mitochondrial damage rapidly induces telomere dysfunction (26). Kumar et al. further proposed a mechanistic link between mitochondrial dysfunction and telomere damage. They suggested that secondary ROS generated by damaged mitochondria may preferentially impair telomere integrity and thereby contribute to cellular senescence and age-related diseases processes (27). Moreover, Tigano et al. reported that mtDNA double-strand breaks activate nuclear immune surveillance pathways (28). Together, these findings suggest that mtDNA damage is not merely a consequence of mitochondrial dysfunction in RIHD. Instead, it may actively drive long-term cardiac remodeling by sustaining ROS production, activating inflammatory responses, and promoting cellular senescence.

### 3.2 Respiratory-chain dysfunction and metabolic reprogramming

Mitochondrial respiratory-chain dysfunction is one of the earliest and most persistent changes in RIHD. Using proteomic analysis, Azimzadeh et al. showed that local cardiac irradiation markedly altered PPARα-related fatty acid metabolic pathways. This finding suggests that dysregulated metabolic nuclear receptor signaling may contribute to the RIHD progression (29). Moreover, a multi-omics study of Mayak nuclear workers linked increased cardiovascular risk to alterations in PPAR signaling, the tricarboxylic acid (TCA) cycle, and glycolysis/gluconeogenesis pathways (30). These findings indicate that radiation-induced mitochondrial injury involves more than ATP depletion. It also causes major changes in substrate utilization and reshapes cellular metabolic network.

Growing evidence suggests that metabolic abnormalities may occur before detectable structural remodeling. Xu et al. showed that radiation-induced disruption of cardiac energy metabolism contributes to subsequent development of tissue fibrosis (31). In addition, Boguszewicz et al. used nuclear magnetic resonance-based metabolomics to analyze mice exposed to low-dose irradiation. They identified significant changes in the cardiac metabolic profile after irradiation (32). Together, these studies suggest that metabolic reprogramming may be a key intermediate process linking early radiation injury to chronic cardiac remodeling.

### 3.3 Mitophagy dysregulation: a critical link between mitochondrial injury and cardiac remodeling

**Figure 2:**
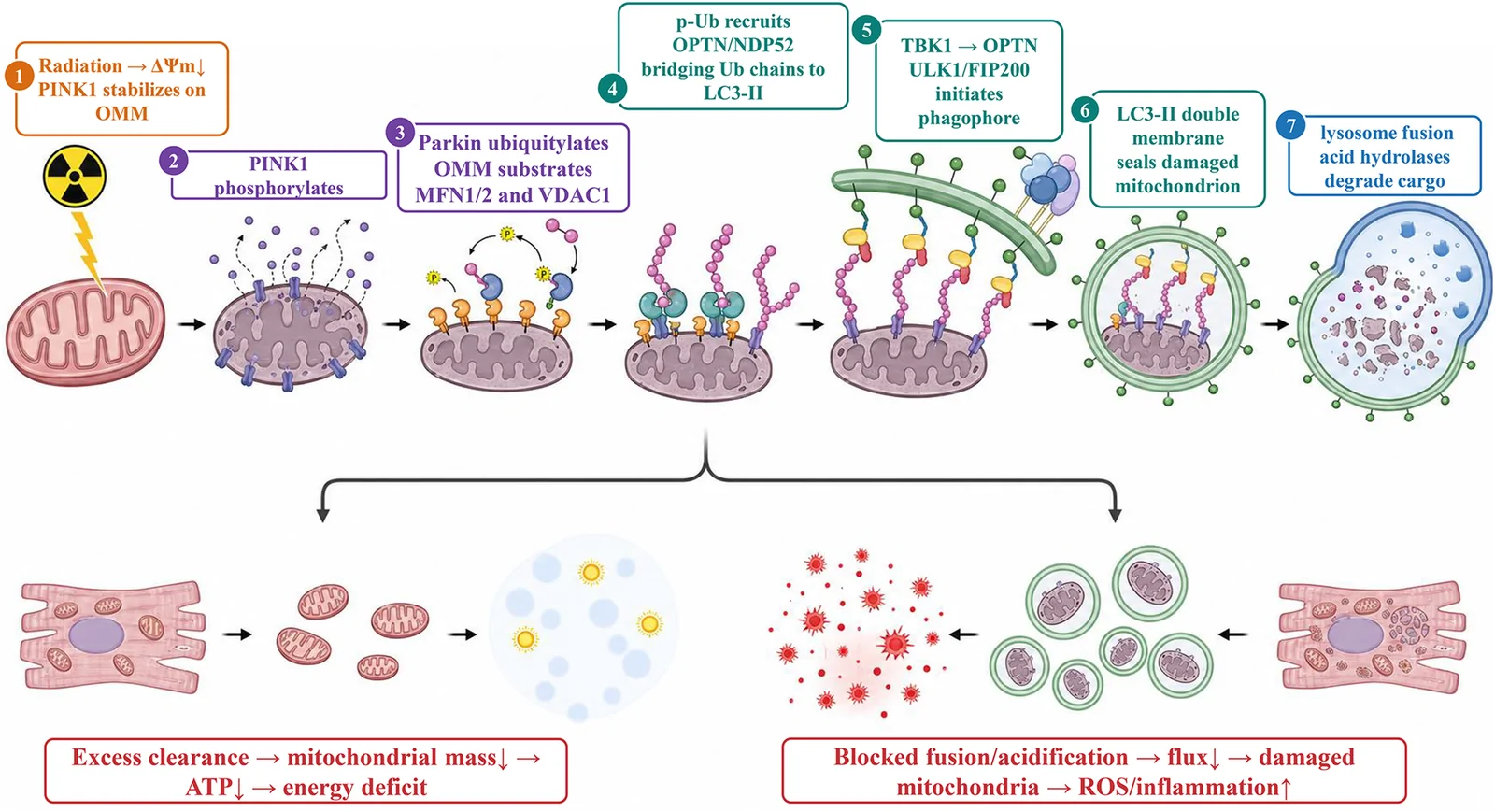
PINK1–Parkin mitophagy mechanism. Radiation-induced loss of mitochondrial membrane potential (ΔΨm) leads to the stabilization of PINK1 on the outer mitochondrial membrane (OMM). PINK1 subsequently phosphorylates ubiquitin and Parkin, activating Parkin to ubiquitinate OMM proteins, including MFN1/2 and VDAC1. The generated phospho-ubiquitin chains recruit autophagy receptors NDP52 and OPTN. Through the TBK1-OPTN-ULK1/FIP200 complex, damaged mitochondria are engulfed by LC3-positive phagophores, which mature into autophagosomes and fuse with lysosomes for degradation. The lower panel illustrates the outcome dichotomy: excessive mitophagy depletes mitochondrial mass and reduces ATP production, whereas impaired lysosomal acidification or fusion blocks autophagic flux, leading to the accumulation of damaged mitochondria, ROS overproduction, and sterile inflammation.

Mitophagy is a selective form of autophagy that removes damaged or unnecessary mitochondria. This process is essential for mitochondrial quality control and cellular energy homeostasis. The PINK1/Parkin pathway is the best-characterized pathway of mitophagy. In healthy mitochondria, PINK1 enters through the TOM and TIM23 complexes. PARL then cleaves PINK1, which is rapidly degraded (33). When the mitochondrial membrane potential declines, PINK1 import and degradation are blocked. As a result, full-length PINK1 accumulates on the outer mitochondrial membrane (34). PINK1 then phosphorylates ubiquitin and Parkin, thereby activating Parkin. Activated Parkin promotes the ubiquitination of outer mitochondrial membrane proteins, including MFN1/2 and VDAC1 (35). The resulting phospho-ubiquitin chains recruit the autophagy receptors NDP52 and OPTN. NDP52 recruits FIP200 and the ULK1 complex to damaged mitochondria. Meanwhile, TBK1 phosphorylates OPTN and strengthens its binding to ubiquitin chains. This process promotes the formation of LC3-positive isolation membranes (36–38). Autophagosomes then engulf the damaged mitochondria and fuse with lysosomes for degradation. In addition to the PINK1/Parkin pathway, FUNDC1 and BNIP3/NIX can bind directly to LC3. These receptors mediate the recognition and removal of damaged mitochondria under hypoxic or metabolic stress (39, 40). Thus, mitophagy is a multistep process that begins with damage recognition and ends with lysosomal degradation. The complete process of the above molecular events is shown in Figure 2.

The role of mitophagy after irradiation depends on two factors: whether damaged mitochondria are completely removed and whether their removal is coordinated with mitochondrial biogenesis. Timely and proportionate regulated mitochondrial clearance helps preserve respiratory function and prevents damaged mitochondria from accumulating (34, 41, 42). However, increased levels of PINK1, Parkin, LC3, or autophagy receptors only indicate changes in mitophagy-related processes. These changes do not prove that mitochondrial degradation has been completed. These proteins may also accumulate when mitochondrial injury persists, autophagosome maturation is delayed, or lysosomal degradation is impaired. In these current evidence situations, damaged mitochondria may remain within the cell despite increased levels of mitophagy-related proteins. Conversely, sustained mitochondrial degradation without sufficient mitochondrial biogenesis may reduce mitochondrial mass and impair ATP production. Therefore, the biological effects of mitophagy depend on the balance among mitochondrial damage, clearance, and replacement.

In cardiac irradiation models, low-dose X-ray exposure increased PINK1/Parkin-related signaling. This increase was accompanied by mitochondrial abnormalities and cardiac hypertrophy (43). Studies of NDP52 and SH3GLB1 further suggest that irradiation may alter the recognition of damaged mitochondria and the assembly of autophagic membranes (9). Nevertheless, most RIHD studies have examined protein expression, molecular interactions, or mitochondrial morphology. Few studies have tracked damaged mitochondria through to lysosomal degradation. Current evidence therefore shows that irradiation alters mitophagy-related signaling, but it does not confirm an increase in complete mitophagic flux. Radiation may continuously generate damaged mitochondria faster than cells can clear and replace them. This imbalance may allow mitochondrial dysfunction and metabolic abnormalities to persist.

Mitophagy dysregulation may depend not only on the abundance of pathway-related proteins but also on how PTMs regulate these proteins. PTMs can alter protein activity, stability, and molecular interactions. Through these effects, PTMs may influence mitochondrial energy metabolism, the recognition of damaged mitochondria, and autophagic membrane formation (44). Studies of RIHD have linked ATP5F1C acetylation (45), NDP52 and SH3GLB1 SUMOylation (9), and P4HB (46) lactylation to changes in mitochondrial metabolism or quality control. These findings suggest that PTMs may form an important regulatory link between radiation-induced stress and persistent dysfunction of mitochondrial quality control.

### 3.4 Distinctive features of mitochondrial dysfunction in RIHD

Mitochondrial pathology in RIHD is fundamentally distinct from that observed in other forms of cardiomyopathy. The primary difference lies in the unique mechanism of radiation injury. Unlike ischemic or pressure-related cardiomyopathies, in which mitochondrial dysfunction often develops secondary to persistent hemodynamic or metabolic stress, radiation can directly damage multiple mitochondrial components during a single exposure event, including mtDNA, respiratory chain proteins, and mitochondrial membranes. Although mitochondrial abnormalities induced by ischemia–reperfusion injury or pressure overload may partially stabilize after the removal of the initiating stress, radiation-induced respiratory chain defects can become a continuous source of ROS leakage. This persistent oxidative stress creates a self-amplifying cycle of mitochondrial injury, leading to the long-term persistence of mitochondrial abnormalities after irradiation (47, 48). Meanwhile, the production of damaged mitochondria does not terminate after irradiation but is continuously promoted by persistent oxidative stress and metabolic reprogramming. In contrast, lysosomal degradative capacity progressively declines in aging cardiomyocytes, creating a state of mitophagy imbalance characterized by enhanced mitochondrial damage recognition but insufficient downstream clearance. The progressive imbalance between mitochondrial damage generation and clearance, rather than a simple increase or decrease in mitochondrial elimination, results in the persistent accumulation of dysfunctional mitochondria in cardiomyocytes. This process ultimately promotes silent and irreversible cardiac remodeling (49). Collectively, these features establish the pathobiological basis of RIHD, which can be characterized as “a single trigger, multiple insults, and continuous progression”. This framework also helps explain the prolonged latency and progressive clinical trajectory of RIHD. This characteristic is particularly relevant in childhood cancer survivors, as radiation exposure often occurs during critical windows of cardiac development and mitochondrial metabolic maturation (50). Consequently, both the initial response of mitochondria to radiation injury and the subsequent long-term consequences may differ from those observed in adult hearts.

### 3.5 Relevance of mitochondrial dysfunction to pediatric RIHD

Therefore, pediatric RIHD warrants special consideration because it involves unique clinical and biological features that differ from those observed in adult hearts. Clinical studies have demonstrated a dose-dependent relationship between cardiac radiation exposure and the subsequent development of heart failure among childhood cancer survivors. Importantly, this risk is determined not only by the mean heart dose but also by the irradiated cardiac volume and the combined exposure to anthracycline chemotherapy (51, 52). Because these complications may remain clinically silent for years, long-term, risk-based cardiac surveillance is recommended for survivors who have received chest irradiation or cardiotoxic chemotherapy (53).

At the mechanistic level, the developing heart cannot simply be regarded as a smaller version of the adult heart. During embryonic and postnatal development, cardiomyocyte mitochondria undergo substantial changes in membrane potential, cristae organization, oxidative capacity, and substrate utilization (54). PINK1–MFN2–Parkin-mediated mitophagy participates in the replacement of fetal mitochondria and the transition from carbohydrate-dependent metabolism toward the oxidative metabolism required by the mature myocardium (10). Radiation-induced mtDNA damage, mitoROS accumulation, or disruption of mitochondrial quality control during this period may therefore affect not only the removal of damaged mitochondria but also normal cardiac metabolic maturation.

However, most available mechanistic evidence comes from adult animals or mature cardiomyocyte models. It remains unclear whether the developing heart responds differently to radiation-induced mitochondrial injury. Future studies should use juvenile irradiation models and compare responses across different ages. Such studies will help clarify how cardiac development influences mitochondrial injury, mitophagy, and subsequent cardiac remodeling after radiation exposure.

## 4 PTM-mediated regulation of mitochondrial targets in RIHD

PTMs can rapidly alter protein activity, stability, subcellular localization, and molecular interactions. They may therefore influence how cells recognize, process, and remove damaged mitochondria after irradiation. Previous reviews have established the role of mitochondrial dysfunction in RIHD. These reviews have focused mainly on mtDNA damage, oxidative stress, transcriptional changes, and apoptosis. However, previous studies have not integrated the emerging evidence on site-specific PTMs in cardiac irradiation models. This review therefore examines ATP5F1C acetylation, NDP52, and SH3GLB1 SUMOylation, and P4HB lactylation. These modifications affect distinct but interconnected aspects of mitochondrial homeostasis, including bioenergetic regulation, damaged-mitochondrial recognition, and autophagic membrane assembly. By integrating these findings, we aim to clarify how PTMs link radiation-induced mitochondrial injury to persistent defects in mitochondrial quality control, while distinguishing mechanisms directly demonstrated in RIHD from those that still require experimental validation.

### 4.1 ATP5F1C acetylation and mitochondrial bioenergetic failure

Acetylation is one of the most extensively studied PTMs in RIHD. The *ATP5F1C* gene encodes the γ-subunit of mitochondrial ATP synthase, which is essential for ATP production. Pecina et al. showed that shRNA-mediated depletion of ATP5F1C disrupted ATP synthase assembly and impaired mitochondrial respiration and ATP production (55). Lu et al. further reported that increased ATP5F1C acetylation was associated with lower ATP levels, loss of mitochondrial membrane potential, and cellular senescence in a high-glucose model. However, the study did not identify the specific modification site (56). In an irradiation model, Barjaktarovic et al. observed widespread hyperacetylation of mitochondrial proteins. This change was accompanied by reduced sirtuin activity and impaired energy metabolism (57). Building on these findings, Zeng et al. used acetylation proteomics to identify K55 as a hyperacetylation site on ATP5F1C. The researchers confirmed this change in irradiated cardiac tissue and cardiomyocytes. Subsequent mutational analysis showed that the acetylation-mimetic K55Q mutant reduced ATP synthase activity and ATP production. The mutant also increased markers of cardiomyocyte senescence (45). Together, these findings suggest that ATP5F1C K55 acetylation may contribute to RIHD by impairing mitochondrial ATP synthesis and promoting cardiomyocyte senescence.

### 4.2 SUMOylation of NDP52 and SH3GLB1 in mitophagy regulation

SUMOylation is a reversible post-translational modification that can regulate cellular stress responses by altering protein–protein interactions (58). NDP52 is an autophagy receptor that connects damaged mitochondria to the autophagic machinery. It recognizes ubiquitin signals on damaged mitochondria and interacts with autophagy-related proteins, including FIP200 and LC3 (34). Using autophagy receptor-knockout cells, Lazarou et al. established the role of NDP52 in recognizing damaged mitochondria (36). Fu et al. later confirmed the direct interaction between NDP52 and FIP200 through structural analysis and *in vitro* binding assays (59). Building on this framework, Gao et al. found that low-dose X-ray irradiation promoted SUMO2 conjugation to NDP52 at K262. The mitochondrial SUMO E3 ligase MUL1 mediated this modification. Compared with wild-type NDP52, the SUMOylation-deficient K262R mutant interacted less strongly with PINK1, LC3, and several autophagy-related proteins. The mutant also showed a reduced ability to recruit damaged mitochondria into the autophagic pathway (43). Thus, K262 SUMOylation appears to enhance the bridging and recruitment function of NDP52 rather than simply increasing its expression. SH3GLB1 may act at a later stage of autophagic membrane assembly. Takahashi et al. used gene deletion, live-cell imaging, and electron microscopy to examine its function. They found that SH3GLB1 deficiency caused immature autophagosomes to accumulate and impaired mitochondrial clearance (60). Gao et al. further reported that irradiation induced SH3GLB1 SUMOylation at K82. This modification strengthened the interactions of SH3GLB1 with LC3, Beclin1, and mitochondrial proteins and contributed to cardiomyocyte hypertrophy (9). These findings suggest that SUMOylation of NDP52 and SH3GLB1 may enhance damaged mitochondrial recruitment and autophagic membrane assembly, respectively. These changes may contribute to impaired mitochondrial quality control in RIHD. However, their effects on complete mitophagic flux remain uncertain.

### 4.3 P4HB lactylation links metabolic reprogramming to mitochondrial quality control

Metabolic reprogramming can increase lactate availability and create the conditions for lysine lactylation, allowing metabolic changes to influence protein function. In hypoxic ovarian cancer, ESM1 promotes PKM2-dependent Warburg metabolism, fatty acid synthesis, and vascular mimicry, thereby establishing a strongly glycolytic metabolic state (61). Within this metabolic setting, ENO1 lactylation further sustains glycolytic activity and angiogenic signaling and contributes to bevacizumab resistance (62). The interaction among lactate accumulation, protein lactylation, metabolic adaptation, and angiogenesis has also been described in other tumor models (63). Whether a comparable metabolism–lactylation relationship contributes to mitochondrial quality-control abnormalities in RIHD remains less clear. Recent findings concerning P4HB provide an initial mechanistic link between these processes.

P4HB, also known as protein disulfide isomerase, is an endoplasmic reticulum chaperone that regulates protein folding and cellular redox homeostasis. Previous studies have linked P4HB-dependent protein interactions to endoplasmic reticulum stress and cardiomyocyte apoptosis. However, these studies did not examine P4HB lactylation. Ouyang et al. combined differential omics analysis with 4D label-free lactylation profiling. They found that X-ray irradiation increased P4HB lactylation. Liquid chromatography–tandem mass spectrometry identified K311 as the principal lactylation site. Site-directed mutagenesis and co-immunoprecipitation further showed that the K311R mutation reduced P4HB lactylation (46). This mutation also weakened the interactions of P4HB with PTGS2, SH3GLB1, NDP52, and LC3. These findings suggest that P4HB K311 lactylation may connect metabolic signals to the mitophagy machinery.

The same study showed that aloe-emodin reduced P4HB K311 lactylation. This effect was accompanied by reduced mitochondrial ROS accumulation, less disruption of kynurenine metabolism, and attenuated cardiac injury. P4HB may therefore act as a key regulatory node that links radiation-induced metabolic reprogramming and lactylation to mitochondrial quality control. However, current evidence for this mechanism relies largely on a single preclinical study. Further independent studies are needed to establish the causal relationship between P4HB lactylation and downstream PTGS2/SH3GLB1/NDP52 signaling (46).

**Figure 3:**
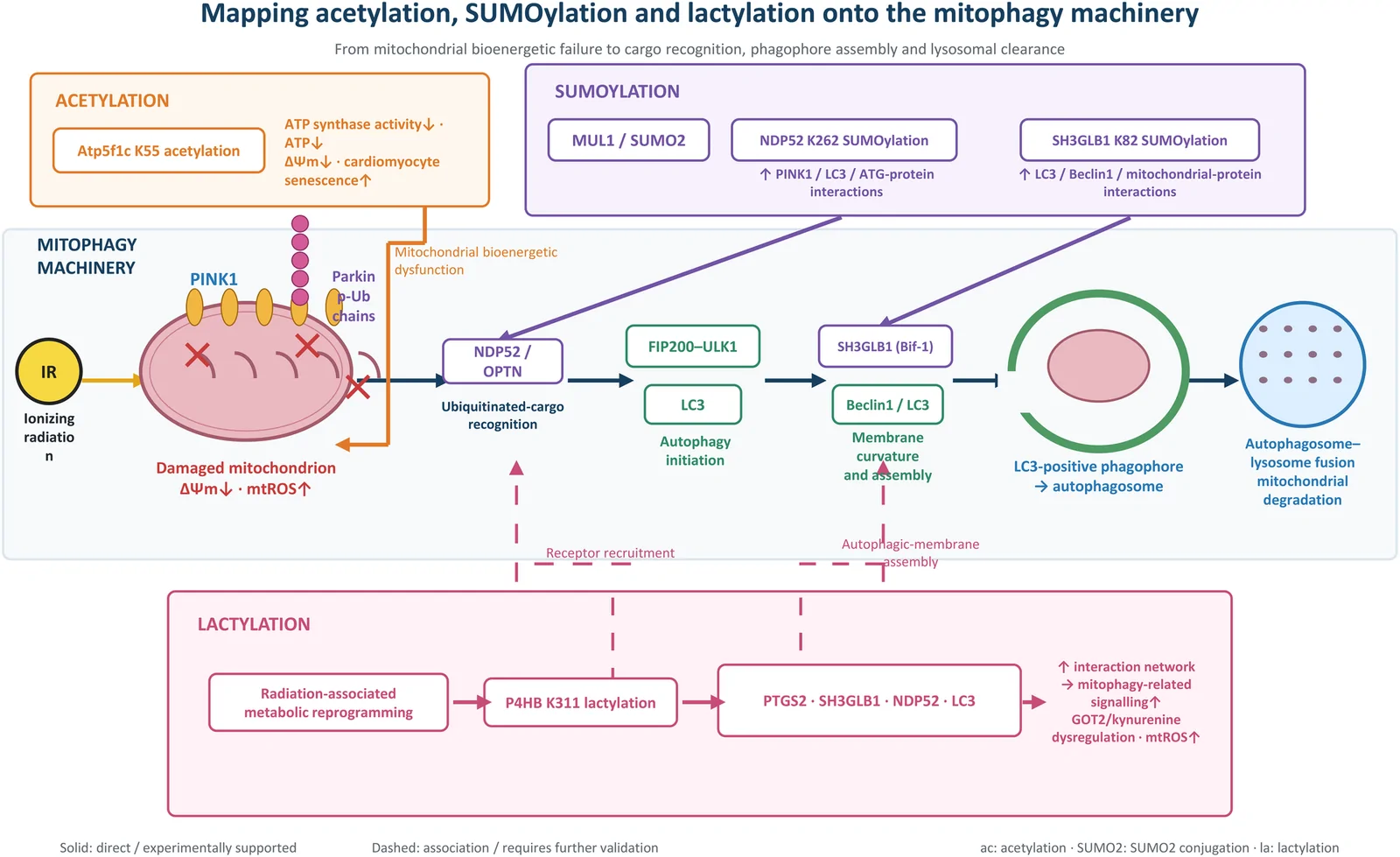
Post-translational modifications and mitophagy. The schematic illustrates the distinct yet interconnected roles of post-translational modifications in targeting mitochondrial components during RIHD. Acetylation of ATP5F1C suppresses ATP synthase activity, impairing bioenergetics and promoting cardiomyocyte senescence. SUMOylation of NDP52 and SH3GLB1 modulates the recognition of damaged mitochondria and the assembly of autophagic membranes, respectively. Lactylation of P4HB links metabolic reprogramming to the mitophagy machinery by facilitating interactions with PTGS2, SH3GLB1, and NDP52. Additionally, the phosphorylation status of the mitophagy receptor FUNDC1 regulates its binding to LC3 and connects mitophagy with mitochondrial fission/fusion dynamics.

These PTMs act at different but interconnected stages of mitochondrial metabolism and quality control, as illustrated in Figure 3.

### 4.4 Phosphorylation-dependent regulation of FUNDC1

FUNDC1 is a mitophagy receptor located on the mitochondrial membrane. Phosphorylation regulates its interaction with LC3. Using FUNDC1 knockdown and reconstitution experiments, Liu et al. showed that FUNDC1 mediates hypoxia-induced mitochondrial clearance through its LC3-interacting region (39). Wu et al. used kinase assays and site-directed mutagenesis to show that ULK1-mediated phosphorylation of FUNDC1 at Ser17 strengthens its binding to LC3. Chen et al. further found that CK2-mediated phosphorylation at Ser13 inhibits the FUNDC1–LC3 interaction. In contrast, PGAM5-mediated dephosphorylation at Ser13 promotes FUNDC1-dependent mitophagy (64).

Using co-immunoprecipitation, Chen et al. showed that the phosphorylation state of FUNDC1 regulates its interactions with DRP1 and OPA1.This mechanism links mitophagy to mitochondrial fission and fusion (65). Using cardiomyocyte-specific *Fundc1*-knockout mice, Wu et al. further showed that FUNDC1 regulates mitochondria–endoplasmic reticulum contacts and Ca^2+^ transfer. These functions help maintain normal cardiac function. These mechanisms may be relevant to radiation-induced mitochondrial ROS accumulation, Ca^2+^ imbalance, and altered mitochondrial dynamics (66). However, researchers have not directly validated them in cardiac irradiation models. FUNDC1 phosphorylation therefore provides a clear mechanistic framework for studying receptor-mediated mitophagy in RIHD. Future studies should confirm its role using irradiation models and phosphosite-specific interventions.

### 4.5 Mechanistic integration and current limitations

Overall, these PTMs act at distinct stages of mitochondrial metabolism and quality control. They regulate mitochondrial energy production, damaged mitochondrial recognition, autophagic membrane assembly, and receptor-mediated mitochondrial clearance. Current evidence links modifications of ATP5F1C, NDP52, SH3GLB1, and P4HB to disrupted mitochondrial homeostasis after irradiation. Meanwhile, FUNDC1 provides a well-established cardiovascular framework for understanding receptor-mediated mitophagy. Therefore, PTMs may help determine whether damaged mitochondria are promptly recognized, removed, and replaced. They should not be viewed merely as secondary molecular changes after radiation exposure.

However, the current evidence has several limitations. Studies of ATP5F1C, NDP52, SH3GLB1, and P4HB have mainly used irradiated cells or rodent models. Most studies have also used single-dose or low-dose irradiation, which does not fully reproduce the fractionated radiotherapy used in clinical practice. In addition, many studies have measured protein expression, pathway markers, or molecular interactions at only a few time points. These approaches cannot reliably distinguish mitophagy initiation, autophagosome accumulation, and completion of lysosomal degradation. Future studies should combine site-specific PTM manipulation with cell type-specific genetic models and dynamic assays of mitophagic flux. They should also use fractionated irradiation protocols that more closely reflect clinical practice. Such studies will help determine whether specific PTMs directly promote cardiac injury, support adaptive protection, or arise secondarily during RIHD progression (Table 1).

**Table 1:** Mitochondria-related candidate therapeutic targets and their regulatory mechanisms in RIHD.

Target	Regulatory mechanism (PTM)	Functional role in mitochondrial homeostasis	Evidence in RIHD/Cardiac Models	Ref.
ATP5F1C	Acetylation (K55)	ATP synthase subunit; acetylation at K55 suppresses ATP production and induces cardiomyocyte senescence.	Elevated acetylation detected in irradiated mouse hearts; K55Q acetylation-mimetic mutant reproduces metabolic dysfunction.	(45)
NDP52(CALCOCO2)	SUMOylation (K262)	Autophagy receptor in PINK1/Parkin-mediated mitophagy; recruits ULK1 complex to damaged mitochondria.	SUMOylation enhances interaction with PINK1/LC3; mediates low-dose X-ray-induced cardiac hypertrophy.	(43)
SH3GLB1	SUMOylation (K82)	Involved in autophagosome formation and membrane scission; interacts with Beclin1.	SUMO2-mediated modification promotes mitophagy activation and hypertrophic cardiomyopathy post-irradiation.	(9)
P4HB	Lactylation (K311)	Endoplasmic reticulum chaperone; connects metabolic signals (kynurenine pathway) to mitophagy machinery.	Lactylation promotes interaction with PTGS2/NDP52; aloe-emodin inhibits this modification to alleviate RIHD.	(46)
FUNDC1	Phosphorylation (S17/S13)	Outer mitochondrial membrane receptor; mediates hypoxia-induced mitophagy via LC3-interacting region.	Mechanistically relevant to mitoROS and Ca^2+^ disturbance in irradiated hearts; direct validation in RIHD models is required.	(39, 65)

## 5 Traditional Chinese medicine and natural products targeting mitochondrial homeostasis: emerging therapeutic strategies for RIHD

Current clinical management of RIHD focuses mainly on optimizing radiotherapy planning, limiting cardiac radiation exposure, and controlling conventional cardiovascular risk factors (67). However, few effective treatments can reverse myocardial fibrosis and structural remodeling once these changes have developed. Therefore, early interventions that preserve mitochondrial homeostasis have become an important focus of RIHD research. Compared with single-target agents, bioactive compounds derived from traditional Chinese medicine and other natural sources often act on multiple targets and signaling pathways. These compounds may simultaneously regulate oxidative stress, energy metabolism, inflammatory, endoplasmic reticulum stress, mitophagy, and PTMs. Their broad biological effects makes them promising candidates for addressing the complex pathological mechanisms of RIHD.

Astragaloside IV (AS-IV) is one of the most widely studied TCM-derived compounds in RIHD. Ren et al. evaluated AS-IV in rats exposed to a single 40-Gy dose of localized thoracic X-ray irradiation. AS-IV reduced serum levels of NT-pro BNP, cTnI, and CK-MB. It also decreased cardiomyocyte apoptosis and collagen deposition. Moreover, AS-IV increased myocardial ATP content and enhanced the activities of mitochondrial respiratory-chain complexes I-IV. It also alleviated mitochondrial Ca^2+^ overload, swelling, cristae disruption, and vacuolization (68). At the signaling level, AS-IV reduced the radiation-induced increases in GPR35 and phosphorylated AMPK and restored phosphorylated mTOR levels. It also decreased the expression of Bax, caspase-3, caspase-9, collagen I, and α-smooth muscle actin. These findings suggest that AS-IV may alleviate radiation-induced myocardial fibrosis by improving mitochondrial energy metabolism and suppressing mitochondria-mediated apoptosis through GPR35/AMPK/mTOR-related signaling. However, the study used a single high dose of radiation and did not validate this pathway through genetic or pharmacological interventions. Therefore, future studies should confirm the proposed mechanism using clinically relevant fractionated irradiation models.

Naringin exerts anti-inflammatory and metabolic regulatory effects. Liu et al. showed that local cardiac X-ray irradiation caused dose-dependent cardiac dysfunction and fibrosis in rats. In the 20-Gy group, naringin improved left ventricular ejection fraction and left ventricular fractional shortening. It also reduced collagen deposition and decreased the expression of collagen I, α-smooth muscle actin, and transforming growth factor-β1 (69). Naringin also reduced the expression of endoplasmic reticulum stress markers, including GRP78, ATF6, CHOP, and caspase-12, restored SIRT1 expression, and suppressed NF-κB p65 phosphorylation. These effects were accompanied by lower levels of IL-6, IL-1beta, MCP-1, and malondialdehyde, together with restored superoxide dismutase and catalase activities. These results suggest that naringin may protect the irradiated heart by reducing endoplasmic reticulum stress, lipid peroxidation, and NF-κB-mediated inflammation. SIRT1 restoration may contribute to these protective effects.

In recent years, therapeutic approaches targeting PTMs have begun to emerge. Using an X-ray-induced RIHD model, Ouyang et al. showed that aloe-emodin improved cardiac function. It also reduced myocardial fibrosis, inflammation, and mitochondrial structural damage (46). The authors combined differential omics analysis with 4D label-free lactylation profiling. They identified P4HB as a radiation-responsive lactylated protein and K311 as its principal modification site. The K311R mutation reduced P4HB lactylation and weakened its interactions with PTGS2, SH3GLB1, NDP52, and LC3. These findings link P4HB lactylation to the mitophagy machinery. Aloe-emodin inhibited P4HB K311 lactylation and reduced the interaction between PTGS2 and SH3GLB1. It also decreased mitoROS accumulation, attenuated NDP52-related mitophagy signaling, and limited the abnormal accumulation of GOT2 on damaged mitochondria. These effects were accompanied by improved kynurenine metabolism (46). This study combined omics-based screening with site-specific mutational analysis and provided systematic evidence for the proposed mechanism. However, independent studies are still needed to confirm these findings. Future research should also use human cardiac models and clinically relevant fractionated irradiation protocols to assess the robustness and clinical relevance of this mechanism.

Overall, research on TCM-derived compounds and natural products in RIHD is gradually moving beyond the traditional concept of “antioxidant protection”. Current studies increasingly focus on restoring mitochondrial and cellular stress homeostasis. AS-IV mainly improves respiratory-chain activity and ATP production. Naringin reduces endoplasmic reticulum stress, oxidative injury, and inflammation. Aloe-emodin links metabolic reprogramming to mitochondrial quality control by regulating P4HB lactylation. Current studies provide preliminary evidence that these bioactive compounds may help prevent or treat RIHD. However, most evidence remains preclinical and is insufficient to support their clinical use. Future studies should use fractionated irradiation protocols that better reflect clinical radiotherapy and should evaluate preventive and post-irradiation treatment separately. Before clinical translation, researchers must also assess the pharmacokinetics and long-term safety of these compounds and determine whether they interfere with the antitumor effects of radiotherapy.

## 6 Discussion

The available evidence supports a model in which mitochondrial injury, altered PTM signaling, and impaired mitochondrial quality control jointly drive the progression of RIHD. Importantly, these processes appear to form a self-reinforcing network rather than acting as independent pathological events. Current evidence indicates that radiation-induced mtDNA damage, respiratory-chain dysfunction, and metabolic reprogramming can sustain mitochondrial stress. Persistent mitochondrial stress may then promote inflammation, cellular senescence, and adverse cardiac remodeling. At the same time, impaired recognition and removal of damaged mitochondria may further disrupt cellular energy homeostasis. Recent studies have also shown that acetylation, SUMOylation, and lactylation may contribute to RIHD by regulating mitochondrial metabolism and the functions of mitophagy-related proteins. These findings suggest that PTMs are not merely secondary changes associated with radiation injury. Instead, they may help determine whether mitochondrial damage is repaired or progresses to persistent cardiac remodeling.

This framework provides a mechanistic link between early radiation-induced mitochondrial injury and delayed cardiac remodeling. It also suggests that the long latency of RIHD may reflect the gradual failure of mitochondrial repair, clearance, and metabolic adaptation. The mitochondria–PTM–mitophagy framework links early mitochondrial injury to subsequent cardiac remodeling. It may help explain the long latency and progressive course of RIHD. This framework may also support the identification of earlier windows for therapeutic intervention.

Current evidence has several important limitations. Studies of ATP5F1C acetylation, NDP52 and SH3GLB1 SUMOylation, and P4HB lactylation have relied mainly on irradiated cell systems and rodent models. Current models do not fully capture the temporal and spatial complexity of clinical radiotherapy. They also provide insufficient evidence to determine whether damaged mitochondria undergo complete lysosomal degradation.

Future research should address these gaps by prioritizing clinical relevance and age-specific effects. Researchers should also determine whether candidate interventions compromise the antitumor efficacy of radiotherapy. Pediatric RIHD requires particular attention because radiation exposure occurs during active cardiac development and mitochondrial maturation. The developing myocardium differs from the adult heart in mitochondrial biogenesis, metabolic flexibility, and mitochondrial quality-control capacity. Therefore, mechanisms identified in adult models may not fully explain the long-term effects of childhood radiation exposure. Future studies should use age-appropriate irradiation models to determine how developmental stage influences mitochondrial injury, PTM-mediated regulation, and mitophagy. Long-term studies of childhood cancer survivors should also examine whether early mitochondrial alterations contribute to cardiovascular complications that emerge decades after treatment. Together, these approaches may facilitate the translation of mechanistic findings into age-specific risk assessment and intervention strategies.

## References

[1] Darby SC Ewertz M McGale P Bennet AM Blom-Goldman U Brønnum D. Risk of ischemic heart disease in women after radiotherapy for breast cancer. N Engl J Med. (2013) 368:987–98. 10.1056/NEJMoa1209825 23484825

[2] van Nimwegen FA Schaapveld M Janus CPM Krol ADG Petersen EJ Raemaekers JMM. Cardiovascular disease after Hodgkin lymphoma treatment. JAMA Intern Med. (2015) 175:1007. 10.1001/jamainternmed.2015.1180 25915855

[3] Richardson DB Leuraud K Laurier D Gillies M Haylock R Kelly-Reif K. Cancer mortality after low dose exposure to ionising radiation in workers in France, the United Kingdom, and the United States (INWORKS): cohort study. BMJ. (2023) 382:e074520. 10.1136/bmj-2022-074520 37586731 PMC10427997

[4] Shimizu Y Kodama K Nishi N Kasagi F Suyama A Soda M. Radiation exposure and circulatory disease risk: Hiroshima and Nagasaki atomic bomb survivor data, 1950–2003. BMJ. (2010) 340:b5349. 10.1136/bmj.b5349 20075151 PMC2806940

[5] Cheng Y-J Nie X-Y Ji C-C Lin X-X Liu L-J Chen X-M. Long-term cardiovascular risk after radiotherapy in women with breast cancer. J Am Heart Assoc. (2017) 6:e005633. 10.1161/JAHA.117.005633 28529208 PMC5524103

[6] Ping Z Peng Y Lang H Xinyong C Zhiyi Z Xiaocheng W. Oxidative stress in radiation-induced cardiotoxicity. Oxid Med Cell Longev. (2020) 2020:1–15. 10.1155/2020/3579143 PMC7071808 32190171

[7] Wang B Wang H Zhang M Ji R Wei J Xin Y. Radiation-induced myocardial fibrosis: mechanisms underlying its pathogenesis and therapeutic strategies. J Cell Mol Med. (2020) 24:7717–29. 10.1111/jcmm.15479 32536032 PMC7348163

[8] Wang K-X Ye C Yang X Ma P Yan C Luo L. New insights into the understanding of mechanisms of radiation-induced heart disease. Curr Treat Options Oncol. (2023) 24:12–29. 10.1007/s11864-022-01041-4 36598620

[9] Gao A Zou J Mao Z Zhou H Zeng G. SUMO2-mediated SUMOylation of SH3GLB1 promotes ionizing radiation-induced hypertrophic cardiomyopathy through mitophagy activation. Eur J Pharmacol. (2022) 924:174980. 10.1016/j.ejphar.2022.174980 35487252

[10] Gong G Song M Csordas G Kelly DP Matkovich SJ Dorn GW. Parkin-mediated mitophagy directs perinatal cardiac metabolic maturation in mice. Science. (2015) 350:aad2459. 10.1126/science.aad2459 26785495 PMC4747105

[11] Murphy E Ardehali H Balaban RS DiLisa F Dorn GW Kitsis RN. Mitochondrial function, biology, and role in disease. Circ Res. (2016) 118:1961–91. 10.1161/RES.0000000000000104 PMC6398603 27126807

[12] MericskayM ZuurbierCJ HeatherLC KarlstaedtA InserteJ BertrandL. Cardiac intermediary metabolism in heart failure: substrate use, signalling roles and therapeutic targets. Nat Rev Cardiol. (2025) 22:704–27. 10.1038/s41569-025-01166-740544173

[13] Rossini M Filadi R. Sarcoplasmic reticulum-mitochondria kissing in cardiomyocytes: Ca^2+^, ATP, and undisclosed secrets. Front Cell Dev Biol. (2020) 8:532. 10.3389/fcell.2020.00532 32671075 PMC7332691

[14] Zuo J Zhang Z Luo M Zhou L Nice EC Zhang W. Redox signaling at the crossroads of human health and disease. MedComm. (2022) 3:e127. 10.1002/mco2.127 35386842 PMC8971743

[15] Piquereau J Caffin F Novotova M Lemaire C Veksler V Garnier A. Mitochondrial dynamics in the adult cardiomyocytes: which roles for a highly specialized cell? Front Physiol. (2013) 4:102. 10.3389/fphys.2013.00102 23675354 PMC3650619

[16] Vásquez-Trincado C García-Carvajal I Pennanen C Parra V Hill JA Rothermel BA. Mitochondrial dynamics, mitophagy and cardiovascular disease. J Physiol. (2016) 594:509–25. 10.1113/JP271301 26537557 PMC5341713

[17] Jin J-Y Wei X-X Zhi X-L Wang X-H Meng D. Drp1-dependent mitochondrial fission in cardiovascular disease. Acta Pharmacol Sin. (2021) 42:655–64. 10.1038/s41401-020-00518-y 32913266 PMC8115655

[18] Alexeyev M Shokolenko I Wilson G LeDoux S. The maintenance of mitochondrial DNA integrity–critical analysis and update. Cold Spring Harbor Perspect Biol. (2013) 5:a012641. 10.1101/cshperspect.a012641 PMC3632056 23637283

[19] Kam WW-Y Banati RB. Effects of ionizing radiation on mitochondria. Free Radical Biol Med. (2013) 65:607–19. 10.1016/j.freeradbiomed.2013.07.024 23892359

[20] Azzam EI Jay-Gerin J-P Pain D. Ionizing radiation-induced metabolic oxidative stress and prolonged cell injury. Cancer Lett. (2012) 327:48–60. 10.1016/j.canlet.2011.12.012 22182453 PMC3980444

[21] Barjaktarovic Z Schmaltz D Shyla A Azimzadeh O Schulz S Haagen J. Radiation–induced signaling results in mitochondrial impairment in mouse heart at 4 weeks after exposure to X-rays. PLoS One. (2011) 6:e27811. 10.1371/journal.pone.0027811 22174747 PMC3234240

[22] Barjaktarovic Z Shyla A Azimzadeh O Schulz S Haagen J Dörr W. Ionising radiation induces persistent alterations in the cardiac mitochondrial function of C57BL/6 mice 40weeks after local heart exposure. Radiother Oncol. (2013) 106:404–10. 10.1016/j.radonc.2013.01.017 23522698

[23] Sridharan V Aykin-Burns N Tripathi P Krager KJ Sharma SK Moros EG. Radiation-induced alterations in mitochondria of the rat heart. Radiat Res. (2014) 181:324. 10.1667/RR13452.1 24568130 PMC4029615

[24] Zhong F Liang S Zhong Z. Emerging role of mitochondrial DNA as a major driver of inflammation and disease progression. Trends Immunol. (2019) 40:1120–33. 10.1016/j.it.2019.10.008 31744765

[25] Philipp J Le Gleut R von Toerne C Subedi P Azimzadeh O Atkinson MJ. Radiation response of human cardiac endothelial cells reveals a central role of the cGAS-STING pathway in the development of inflammation. Proteomes. (2020) 8:30. 10.3390/proteomes8040030 33114474 PMC7709117

[26] Qian W Kumar N Roginskaya V Fouquerel E Opresko PL Shiva S. Chemoptogenetic damage to mitochondria causes rapid telomere dysfunction. Proc Natl Acad Sci U S A. (2019) 116:18435–44. 10.1073/pnas.1910574116 31451640 PMC6744920

[27] Kumar N Qian W Van Houten B. Sick mitochondria cause telomere damage: implications for disease. Mol Cell Oncol. (2020) 7:1678362. 10.1080/23723556.2019.1678362 31993494 PMC6961657

[28] Tigano M Vargas DC Tremblay-Belzile S Fu Y Sfeir A. Nuclear sensing of breaks in mitochondrial DNA enhances immune surveillance. Nature. (2021) 591:477–81. 10.1038/s41586-021-03269-w 33627873

[29] Azimzadeh O Sievert W Sarioglu H Yentrapalli R Barjaktarovic Z Sriharshan A. PPAR alpha: a novel radiation target in locally exposed Mus musculus heart revealed by quantitative proteomics. J Proteome Res. (2013) 12:2701–14. 10.1021/pr400071g 23560462

[30] Papiez A Azimzadeh O Azizova T Moseeva M Anastasov N Smida J. Integrative multiomics study for validation of mechanisms in radiation-induced ischemic heart disease in Mayak workers. PLoS One. (2018) 13:e0209626. 10.1371/journal.pone.0209626 30596717 PMC6312255

[31] Xu P Yi Y Luo Y Liu Z Xu Y Cai J. Radiation-induced dysfunction of energy metabolism in the heart results in the fibrosis of cardiac tissues. Mol Med Rep. (2021) 24:842. 10.3892/mmr.2021.12482 34633055 PMC8524410

[32] Gramatyka M Boguszewicz ᴌ Ciszek M Gabryś D Kulik R Sokół M. Metabolic changes in mice cardiac tissue after low-dose irradiation revealed by 1H NMR spectroscopy. J Radiat Res. (2020) 61:14–26. 10.1093/jrr/rrz079 31840756 PMC6976729

[33] Jin SM Lazarou M Wang C Kane LA Narendra DP Youle RJ. Mitochondrial membrane potential regulates PINK1 import and proteolytic destabilization by PARL. J Cell Biol. (2010) 191:933–42. 10.1083/jcb.201008084 21115803 PMC2995166

[34] Onishi M Yamano K Sato M Matsuda N Okamoto K. Molecular mechanisms and physiological functions of mitophagy. EMBO J. (2021) 40:EMBJ2020104705. 10.15252/embj.2020104705 PMC7849173 33438778

[35] Koyano F Okatsu K Kosako H Tamura Y Go E Kimura M. Ubiquitin is phosphorylated by PINK1 to activate parkin. Nature. (2014) 510:162–6. 10.1038/nature13392 24784582

[36] Lazarou M Sliter DA Kane LA Sarraf SA Wang C Burman JL. The ubiquitin kinase PINK1 recruits autophagy receptors to induce mitophagy. Nature. (2015) 524:309–14. 10.1038/nature14893 26266977 PMC5018156

[37] Heo J-M Ordureau A Paulo JA Rinehart J Harper JW. The PINK1-PARKIN mitochondrial ubiquitylation pathway drives a program of OPTN/NDP52 recruitment and TBK1 activation to promote mitophagy. Mol Cell. (2015) 60:7–20. 10.1016/j.molcel.2015.08.016 26365381 PMC4592482

[38] Moore AS Holzbaur ELF. Dynamic recruitment and activation of ALS-associated TBK1 with its target optineurin are required for efficient mitophagy. Proc Natl Acad Sci U S A. (2016) 113:E3349–58. 10.1073/pnas.1523810113 27247382 PMC4914160

[39] Liu L Feng D Chen G Chen M Zheng Q Song P. Mitochondrial outer-membrane protein FUNDC1 mediates hypoxia-induced mitophagy in mammalian cells. Nat Cell Biol. (2012) 14:177–85. 10.1038/ncb2422 22267086

[40] Marinković M Novak I. A brief overview of BNIP3L/NIX receptor-mediated mitophagy. FEBS Open Bio. (2021) 11:3230–6. 10.1002/2211-5463.13307 PMC8634856 34597467

[41] Bravo-San Pedro JM Kroemer G Galluzzi L. Autophagy and mitophagy in cardiovascular disease. Circ Res. (2017) 120:1812–24. 10.1161/CIRCRESAHA.117.311082 28546358

[42] Morciano G Patergnani S Bonora M Pedriali G Tarocco A Bouhamida E. Mitophagy in cardiovascular diseases. J Clin Med. (2020) 9:892. 10.3390/jcm9030892 32214047 PMC7141512

[43] Gao A Wang M Tang X Shi G Hou K Fang J. NDP52 SUMOylation contributes to low-dose X-rays-induced cardiac hypertrophy through PINK1/Parkin-mediated mitophagy via MUL1/SUMO2 signalling. J Cell Physiol. (2024) 239:79–96. 10.1002/jcp.31145 37942585

[44] Wang L Qi H Tang Y Shen H-M. Post-translational modifications of key machinery in the control of mitophagy. Trends Biochem Sci. (2020) 45:58–75. 10.1016/j.tibs.2019.08.002 31606339

[45] Zeng Z Xu P He Y Yi Y Liu Z Cai J. Acetylation of Atp5f1c mediates cardiomyocyte senescence via metabolic dysfunction in radiation-induced heart damage. Oxid Med Cell Longev. (2022) 2022:4155565. 10.1155/2022/4155565 36160705 PMC9499811

[46] Ouyang F Li Y Wang H Liu X Tan X Xie G. Aloe emodin alleviates radiation-induced heart disease via blocking P4HB lactylation and mitigating kynurenine metabolic disruption. Adv Sci (Weinh). (2024) 11:2406026. 10.1002/advs.202406026 39494721 PMC11653682

[47] Yang H-M. Mitochondrial dysfunction in cardiovascular diseases. Int J Mol Sci. (2025) 26:1917. 10.3390/ijms26051917 40076543 PMC11900462

[48] Livingston K Schlaak RA Puckett LL Bergom C. The role of mitochondrial dysfunction in radiation-induced heart disease: from bench to bedside. Front Cardiovasc Med. (2020) 7:20. 10.3389/fcvm.2020.00020 32154269 PMC7047199

[49] Dutta D Calvani R Bernabei R Leeuwenburgh C Marzetti E. Contribution of impaired mitochondrial autophagy to cardiac aging: mechanisms and therapeutic opportunities. Circ Res. (2012) 110:1125–38. 10.1161/CIRCRESAHA.111.246108 22499902 PMC3353545

[50] Leerink JM de Baat EC Feijen EAM Bellersen L van Dalen EC Grotenhuis HB. Cardiac disease in childhood cancer survivors: risk prediction, prevention, and surveillance: JACC CardioOncology state-of-the-art review. JACC CardioOncol. (2020) 2:363–78. 10.1016/j.jaccao.2020.08.006 34396245 PMC8352294

[51] de Baat EC Feijen EAM Reulen RC Allodji RS Bagnasco F Bardi E. Risk factors for heart failure among pan-European childhood cancer survivors: a PanCareSurFup and ProCardio cohort and nested case-control study. J Clin Oncol. (2023) 41:96–106. 10.1200/JCO.21.02944 36075007 PMC9788976

[52] Bates JE Rancati T Keshavarz H Gagliardi G Aznar MC Howell RM. Cardiac disease in childhood cancer survivors treated with radiation therapy: a PENTEC comprehensive review. Int J Radiat Oncol Biol Phys. (2024) 119:522–32. 10.1016/j.ijrobp.2023.03.045 37061912

[53] Armenian SH Hudson MM Mulder RL Chen MH Constine LS Dwyer M. Recommendations for cardiomyopathy surveillance for survivors of childhood cancer: a report from the international late effects of childhood cancer guideline harmonization group. Lancet Oncol. (2015) 16:e123–36. 10.1016/S1470-2045(14)70409-7 25752563 PMC4485458

[54] Hom JR Quintanilla RA Hoffman DL de Mesy Bentley KL Molkentin JD Sheu S-S. The permeability transition pore controls cardiac mitochondrial maturation and myocyte differentiation. Dev Cell. (2011) 21:469–78. 10.1016/j.devcel.2011.08.008 21920313 PMC3175092

[55] Pecina P Nůsková H Karbanová V Kaplanová V Mráček T Houštěk J. Role of the mitochondrial ATP synthase central stalk subunits γ and δ in the activity and assembly of the mammalian enzyme. Biochim Biophys Acta Bioenerg. (2018) 1859:374–81. 10.1016/j.bbabio.2018.02.007 29499186

[56] Lu Q Hu X Hou Q Yu L Cao K Ding D. Rheb1 deficiency elicits mitochondrial dysfunction and accelerates podocyte senescence through promoting Atp5f1c acetylation. Cell Signal. (2024) 124:111451. 10.1016/j.cellsig.2024.111451 39389178

[57] Barjaktarovic Z Merl-Pham J Braga-Tanaka I Tanaka S Hauck SM Saran A. Hyperacetylation of cardiac mitochondrial proteins is associated with metabolic impairment and sirtuin downregulation after chronic total body irradiation of ApoE -/- mice. Int J Mol Sci. (2019) 20:5239. 10.3390/ijms20205239 31652604 PMC6829468

[58] Sahin U De Thé H Lallemand-Breitenbach V. SUMOylation in physiology, pathology and therapy. Cells. (2022) 11:814. 10.3390/cells11050814 35269436 PMC8909597

[59] Fu T Zhang M Zhou Z Wu P Peng C Wang Y. Structural and biochemical advances on the recruitment of the autophagy-initiating ULK and TBK1 complexes by autophagy receptor NDP52. Sci Adv. (2021) 7:eabi6582. 10.1126/sciadv.abi6582 34389544 PMC8363153

[60] Takahashi Y Hori T Cooper TK Liao J Desai N Serfass JM. Bif-1 haploinsufficiency promotes chromosomal instability and accelerates Myc-driven lymphomagenesis via suppression of mitophagy. Blood. (2013) 121:1622–32. 10.1182/blood-2012-10-459826 23287860 PMC3587325

[61] Zhang J Ouyang F Gao A Zeng T Li M Li H. ESM1 enhances fatty acid synthesis and vascular mimicry in ovarian cancer by utilizing the PKM2-dependent warburg effect within the hypoxic tumor microenvironment. Mol Cancer. (2024) 23:94. 10.1186/s12943-024-02009-8 38720298 PMC11077861

[62] Zeng T Wang Q Li L Tan X He J Liu Y. ENO1 lactylation drives bevacizumab resistance through metabolic reprogramming and angiogenesis in ovarian cancer. Drug Resist Updat. (2026) 87:101413. 10.1016/j.drup.2026.101413 42134086

[63] Wu P Zhang J Jiang Z Liu X Jiang N Zou J. Lactate-lactylation in tumor angiogenesis and progression: mechanisms, biomarker potential, and therapeutic implications. Biomarker Res. (2026) 14:21. 10.1186/s40364-026-00898-z PMC12857134 41593811

[64] Chen G Han Z Feng D Chen Y Chen L Wu H. A regulatory signaling loop comprising the PGAM5 phosphatase and CK2 controls receptor-mediated mitophagy. Mol Cell. (2014) 54:362–77. 10.1016/j.molcel.2014.02.034 24746696

[65] Chen M Chen Z Wang Y Tan Z Zhu C Li Y. Mitophagy receptor FUNDC1 regulates mitochondrial dynamics and mitophagy. Autophagy. (2016) 12:689–702. 10.1080/15548627.2016.1151580 27050458 PMC4836026

[66] Wu S Lu Q Wang Q Ding Y Ma Z Mao X. Binding of FUN14 domain containing 1 with inositol 1,4,5-trisphosphate receptor in mitochondria-associated endoplasmic reticulum membranes maintains mitochondrial dynamics and function in hearts in vivo. Circulation. (2017) 136:2248–66. 10.1161/CIRCULATIONAHA.117.030235 28942427 PMC5716911

[67] Cuomo JR Javaheri SP Sharma GK Kapoor D Berman AE Weintraub NL. How to prevent and manage radiation-induced coronary artery disease. Heart. (2018) 104:1647–53. 10.1136/heartjnl-2017-312123 29764968 PMC6381836

[68] Ren C-Z Li Q-R Shu C Chen Q-L Li L-C Jiang H-G. [Astragaloside Ⅳ ameliorates mitochondrial energy metabolism and inhibits radiation-induced myocardial fibrosis in rats via regulating GPR35/AMPK/mTOR signaling axis]. Zhongguo Zhong Yao Za Zhi. (2026) 51:505–14. 10.19540/j.cnki.cjcmm.20251011.707 41814737

[69] Liu S-T Zha K-J Li P-J Gao J-B Zhang Y-G. Protective effect of naringin against radiation-induced heart disease in rats via Sirt1/NF-κB signaling pathway and endoplasmic reticulum stress. Chem Biol Drug Des. (2024) 103:e14453. 10.1111/cbdd.14453 38230793

